# A recalibrated prediction model can identify level-1 trauma patients at risk of nosocomial pneumonia

**DOI:** 10.1007/s00402-023-04766-5

**Published:** 2023-01-17

**Authors:** T. Kobes, A. M. Terpstra, F. F. A. IJpma, L. P. H. Leenen, R. M. Houwert, K. J. P. van Wessem, R. H. H. Groenwold, M. C. P. M. van Baal

**Affiliations:** 1 Department of Trauma Surgery, University Medical Center Utrecht, PO Box 85500, 3508GA Utrecht, The Netherlands; 2 Regional Trauma Network, University Medical Center Utrecht, Utrecht, The Netherlands; 3 Department of Trauma Surgery, University Medical Center Groningen, Groningen, The Netherlands; 4 Department of Clinical Epidemiology, Leiden University Medical Center, Leiden, The Netherlands; 5 Department of Biomedical Data Sciences, Leiden University Medical Center, Leiden, The Netherlands

**Keywords:** Trauma patients, Nosocomial pneumonia, Prediction model, External validation, Recalibration

## Abstract

**Introduction:**

Nosocomial pneumonia has poor prognosis in hospitalized trauma patients. Croce et al. published a model to predict post-traumatic ventilator-associated pneumonia, which achieved high discrimination and reasonable sensitivity. We aimed to externally validate Croce’s model to predict nosocomial pneumonia in patients admitted to a Dutch level-1 trauma center.

**Materials and methods:**

This retrospective study included all trauma patients (≥ 16y) admitted for > 24 h to our level-1 trauma center in 2017. Exclusion criteria were pneumonia or antibiotic treatment upon hospital admission, treatment elsewhere > 24 h, or death < 48 h. Croce’s model used eight clinical variables—on trauma severity and treatment, available in the emergency department—to predict nosocomial pneumonia risk. The model’s predictive performance was assessed through discrimination and calibration before and after re-estimating the model’s coefficients. In sensitivity analysis, the model was updated using Ridge regression.

**Results:**

809 Patients were included (median age 51y, 67% male, 97% blunt trauma), of whom 86 (11%) developed nosocomial pneumonia. Pneumonia patients were older, more severely injured, and underwent more emergent interventions. Croce’s model showed good discrimination (AUC 0.83, 95% CI 0.79–0.87), yet predicted probabilities were too low (mean predicted risk 6.4%), and calibration was suboptimal (calibration slope 0.63). After full model recalibration, discrimination (AUC 0.84, 95% CI 0.80–0.88) and calibration improved. Adding age to the model increased the AUC to 0.87 (95% CI 0.84–0.91). Prediction parameters were similar after the models were updated using Ridge regression.

**Conclusion:**

The externally validated and intercept-recalibrated models show good discrimination and have the potential to predict nosocomial pneumonia. At this time, clinicians could apply these models to identify high-risk patients, increase patient monitoring, and initiate preventative measures. Recalibration of Croce’s model improved the predictive performance (discrimination and calibration). The recalibrated model provides a further basis for nosocomial pneumonia prediction in level-1 trauma patients. Several models are accessible via an online tool.

**Level of evidence:**

Level III, Prognostic/Epidemiological Study.

**Supplementary Information:**

The online version contains supplementary material available at 10.1007/s00402-023-04766-5.

## Background

Nosocomial pneumonia is one of the most common nosocomial infections, with an incidence of 6 to 38% in patients admitted to a level-1 trauma center [2, 5]. In hospitalized trauma patients, nosocomial pneumonia is associated with poor prognoses, such as an increased hospital length of stay, higher in-hospital mortality, and significantly worse neurologic outcomes one year later [7, 8, 17]. In the Netherlands, pneumonia and respiratory insufficiency contribute to 24 percent of late in-hospital deaths in hospitalized trauma patients [12]. Early identification of patients at risk of developing pneumonia might improve outcomes as it offers opportunities for (earlier) antibiotic treatment or antimicrobial prophylaxis. Hence, accurate prediction is needed to decrease mortality, morbidity, and costs potentially.

To identify level-1 trauma patients at risk of post-traumatic pneumonia, Croce et al. studied potential risk factors measured at the Emergency Department (ED) [5]. In a subsequent study, a risk prediction model showed high discriminatory capacity and reasonable concordance for predicting ventilator-associated pneumonia (VAP) [6]. Guidelines generally describe hospital-acquired pneumonia (HAP) as a different entity of nosocomial pneumonia compared to VAP, as mechanical ventilation enables upper respiratory tract colonization [15]. Nonetheless, guideline criteria are not unanimous in the exact delimitations (e.g., duration of mechanical ventilation) between HAP and VAP, complicating the interpretation of VAP in research [1, 11]. Furthermore, in clinical practice, the treatment of both nosocomial pneumonia entities is roughly similar. Therefore, predicting both HAP and VAP using one model is desirable.

Except for Croce’s model for post-traumatic VAP, models predicting HAP or VAP in trauma patients are lacking. Furthermore, no studies have yet externally validated the Croce model. Therefore, this study aims to externally validate the Croce model to predict nosocomial pneumonia in patients admitted to a Dutch level-1 trauma center.

## Methods

### Study design

An external validation and recalibration study of Croce’s model was performed, aiming to predict the risk of nosocomial pneumonia in all patients admitted to a level-1 trauma center after traumatic injury. This retrospective cohort study was conducted at the University Medical Center Utrecht (UMCU), a level-1 trauma center and academic teaching hospital in the Netherlands. The medical ethical committee of our institution approved this study and waived the need for informed consent (protocol number 20–599/C). This study was reported according to the TRIPOD checklist (Supplemental Material).

### Patients

Eligible patients were identified and retrieved from the local trauma registry that is part of the Dutch National Trauma Registry. This registry, collected and monitored by trained data managers and trauma surgeons, includes injured patients (Abbreviation Injury Scale (AIS) score ≥ 2) who visited an ED within 48 h after trauma, directly followed by hospital admission.

The local trauma registry provided all trauma patients (≥ 16 years) admitted to the UMCU in 2017. Exclusion criteria were a hospital stay of < 24 h; community-acquired pneumonia or antibiotic treatment for any indication upon admission; primary treatment for > 24 h in another hospital before transportation to the UMCU. Patients who died within 48 h of admission were excluded, as pneumonia is unlikely to be the cause of death in these cases; this also was an exclusion criterion in the original study by Croce et al. [6]. Follow-up was until hospital discharge, transfer to another hospital, or in-hospital mortality.

### Croce’s prediction model

Croce et al. developed the post-traumatic VAP model using 9721 patients; all admitted patients from a single level-1 trauma center (in Memphis, Tennessee) who survived the first 48 h were included [6]. The outcome of interest was microbiology-confirmed VAP. The model consisted of eight variables, available in the ED or shortly after: mechanism of injury, either blunt or penetrating; Glasgow Coma Scale (GCS) score; the presence of spinal cord injury; AIS score for the thorax; emergent laparotomy (< 24 h); the number of blood products transfused at the ED; Injury Severity Score (ISS); and emergent intubation before ED discharge. The subsequent model is presented in Table 1. Internal validation of the model was performed in 708 patients using the same in- and exclusion criteria. The area-under-the-receiver operating characteristic curve (AUC) was 0.97 at the internal validation.

**Table 1:** Croce’s formulas for posttraumatic ventilator-associated pneumonia prediction [6]

$$\begin{gathered} f\left( x \right) = - 3.08 - 1.56 \left( {MOI} \right) - 0.12 \left( {GCS} \right) + 1.37 \left( {SCI} \right) + 0.30 \left( {AIS \,thorax} \right) \hfill \\ + 1.87 \left( {lap} \right) + 0.67 \left( {tx} \right) + 0.05 \left( {ISS} \right) + 0.66 \left( {int} \right) \hfill \\ \end{gathered}$$	(Formula 1)
$$P{\text{(pneumonia}} = 1{|}x) = {\raise0.7ex\hbox{$1$} \!\mathord{\left/ {\vphantom {1 {1 + e^{ - f\left( x \right)} }}}\right.\kern-0pt} \!\lower0.7ex\hbox{${1 + e^{ - f\left( x \right)} }$}}$$	(Formula 2)

Dichotomous variables: MOI, blunt (0) or penetrating (1); SCI, yes (1) or no (0); lap, yes (1) or no (0); int, yes (1) or no (0)

*AIS* Abbreviated Injury Scale, *GCS* Glasgow Coma Scale score, *int* emergency intubation, *ISS* Injury Severity Score, *lap* emergent laparotomy, *MOI* mechanism of injury, *P*_pneumonia_ predicted probability of pneumonia development, *SCI* spinal cord injury, *tx* number of blood product units administered

### Variables

The local trauma registry provided data on age, gender, ISS, AIS codes for injuries, GCS score, the mechanism of injury, hospital length of stay (time from ED admission to hospital discharge), intensive care unit (ICU) length of stay, and days on mechanical ventilation of all eligible patients. Separately, medical records were reviewed to obtain the remaining variables (i.e., emergent laparotomy, intubation, and the number of blood products in the ED). The clinical predictors were obtained from the ED visit; emergent laparotomy could be collected up to 24 h after the injury.

Spinal cord injury was defined as spinal cord laceration or incomplete or complete cord syndrome. Emergent laparotomy had to be performed within 24 h of ED admission. Packed red blood cells and fresh frozen plasma were considered blood products; only blood products administered in the ED were counted. Emergent intubation was defined as intubation performed before ED discharge (i.e., prehospital or in-hospital).

### Outcomes

The primary outcome of this study was nosocomial pneumonia. This variable and data on other infectious complications were manually extracted from medical records. Other than being absent upon hospital admission, exact criteria for pneumonia diagnosis were unavailable due to the study’s retrospective nature. Patients receiving antibiotic treatment due to clinically suspected pneumonia were considered to suffer from nosocomial pneumonia. The treating physician decided whether to treat patients with antibiotics. HAP and VAP were each scored as well. Patients who (had) received mechanical ventilation in the ICU, regardless of its duration, were considered at risk of VAP. The remaining patients were considered at risk of HAP.

### Statistical analysis

All analyses were performed using R for Mac (© The R Foundation for Statistical Computing, 2019) and necessary additional packages. Variables were described by the median and interquartile range (IQR), or proportion and percentage. Differences between pneumonia cases and patients who did not develop pneumonia were assessed using bivariate (non-)parametric tests for continuous, ordinal, and dichotomous variables. There were no missing data among the predictors. A *p* value < 0.05 was considered significant.

The external validation of the Croce model consisted of two steps. First, the Croce model was applied to all patients in our cohort, providing individual predicted probabilities of pneumonia (Table 1). Based on these probabilities, we: (1) assessed the discrimination of the model by quantifying the AUC (equivalent to the c-statistic) and 95% confidence interval (CI); (2) calculated calibration-in-the-large by comparing the average predicted risk with the observed pneumonia incidence; (3) created a calibration plot and estimated the calibration slope, and (4) calculated the Brier’s statistic to assess the model accuracy.

Second, the model’s intercept was recalibrated to account for a difference in pneumonia incidence between Croce’s cohort and ours. Third, a full model recalibration was performed, in which the model’s intercept and all other regression coefficients were updated. To this end, a logistic regression model was fitted to the data using the same variables as the Croce model. The model’s discrimination, calibration, and accuracy were assessed for the second and third steps.

Additionally, two sensitivity analyses were performed. At first, the Croce model was adjusted by removing the mechanism of injury as a variable; mechanism of injury was removed as the vast majority of injuries in Dutch trauma patients are blunt and penetrating injuries are uncommon. After that, age was included as an additional variable; age was selected since it is widely available, a known risk factor for nosocomial pneumonia, and significantly associated with pneumonia in multivariable analysis in the preparatory study of Croce et al. [2, 4, 5]. Again, both models were fitted using logistic regression analysis. Ridge regression penalization, using tenfold cross-validation, was applied in full model recalibration and the two sensitivity analyses to account for possible overfitting of the data. We assessed discrimination, calibration, and accuracy for all additional models.

The AUCs and Brier’s statistics were calculated for the HAP and VAP subgroups in all validation and recalibration steps to indicate whether selective digestive decontamination influenced pneumonia prediction in ICU patients.

## Results

### Cohort characteristics

**Fig. 1:**
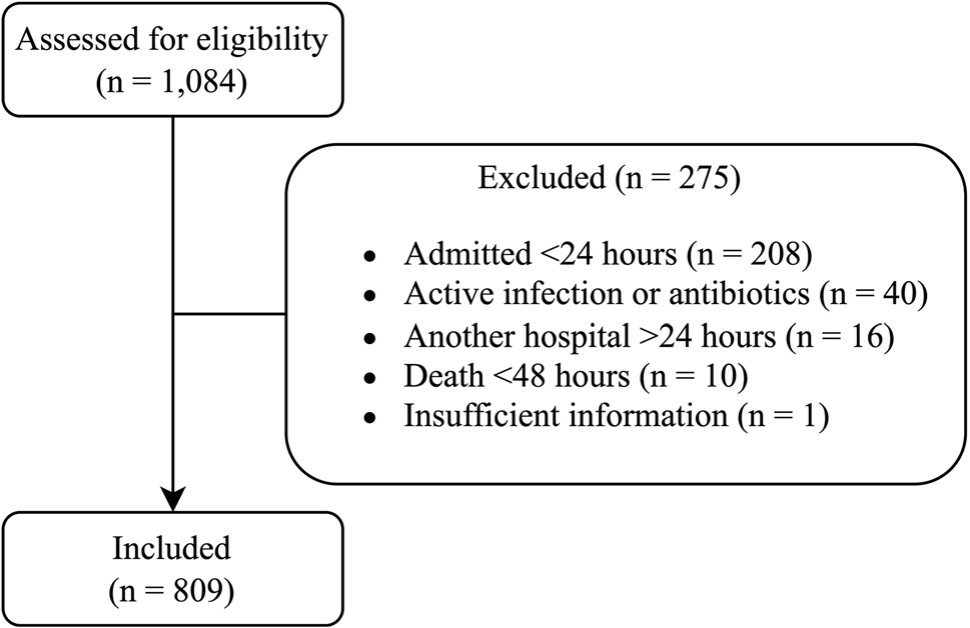
Flowchart of level-1 trauma patient selection for analysis

Out of 1084 eligible patients, 809 patients were included. The 275 patients were excluded based on: admitted to UMCU < 24 h (*n* = 208), active infection or antibiotic treatment upon admission (*n* = 40), initial treatment in another hospital > 24 h (*n* = 16), death < 48 h after admission (*n* = 10), or insufficient information after transfer from another hospital (*n* = 1) (Fig. 1). The median age of the included patients was 51 [IQR 31 – 68] years, 66.9% were male, and 97.2% suffered from blunt trauma. Nosocomial pneumonia incidence was 10.6% (*n* = 86), consisting of 50 cases of VAP (147 patients were at risk of VAP) and 36 cases of HAP (662 patients were at risk of HAP) (Table 2). Other infectious complications (e.g., urinary tract infection, wound infection) occurred in 76 patients (9.4%), of whom 18 also had nosocomial pneumonia.

**Table 2:** Characteristics of the total cohort and subgroups stratified by the occurrence of nosocomial pneumonia

	Total (*n* = 809)	No pneumonia (*n* = 723)	Pneumonia (*n* = 86)	*p* value
Age, median [IQR]	51 [31 – 68]	50 [30 – 66]	60 [44 – 73]	< 0.001^a^
Sex				0.227^b^
Male	541 (67%)	478 (66%)	63 (73%)	
Female	268 (33%)	245 (34%)	23 (27%)	
ASA, median [IQR]	2 [1 – 2]	2 [1 – 2]	2 [1 – 2]	0.03^a^
1–2	693 (86%)	624 (86%)	69 (80%)	
3–4	114 (14%)	97 (13%)	17 (20%)	
Missing	2 (0%)	2 (0%)	0 (0%)	
Mechanism of injury				0.160^c^
Blunt	786 (97%)	700 (97%)	86 (100%)	
MVC	126 (16%)	109 (15%)	17 (20%)	
Bicycle	138 (17%)	119 (17%)	19 (22%)	
Low-energy fall	222 (27%)	207 (29%)	15 (17%)	
High-energy fall	93 (12%)	77 (10%)	16 (19%)	
Other	207 (26%)	188 (26%)	19 (22%)	
Penetrating	23 (3%)	23 (3%)	0 (0%)	
ISS, median [IQR]	10 [5 – 17]	9 [5 – 14]	22 [14 – 29]	< 0.001^a^
< 16	570 (71%)	546 (76%)	24 (28%)	
16 – 25	146 (18%)	120 (17%)	26 (3%)	
> 25	93 (12%)	57 (8%)	36 (42%)	
GCS score, median [IQR]	15 [14 – 15]	15 [14 – 15]	13 [3 – 15]	< 0.001^a^
AIS per body region, median [IQR]				
Head	1 [0 – 2]	0 [0 – 2]	2 [0 – 4]	< 0.001^a^
Spine	0 [0 – 2]	0 [0 – 2]	0 [0 – 2]	< 0.001^a^
Thorax	0 [0 – 2]	0 [0 – 2]	3 [0 – 3]	< 0.001^a^
Abdomen	0 [0 – 0]	0 [0 – 0]	0 [0 – 1]	< 0.001^a^
Spinal cord injury	19 (2%)	15 (2%)	4 (5%)	0.134^c^
Emergent intubation	62 (8%)	46 (6%)	16 (19%)	< 0.001^b^
Administered blood products at ED	41 (5%)	26 (4%)	15 (17%)	< 0.001^b^
If yes, number of units, median [IQR]	2 [2 – 4]	2 [2 – 4]	2 [2 – 3]	< 0.001^a^
Emergent laparotomy	26 (3%)	20 (3%)	6 (7%)	< 0.077^b^
HLOS, median [IQR]	5 [2 – 11]	5 [2 – 9]	17 [11 – 30]	< 0.001^a^
ICU admitted patients	163 (20%)	105 (15%)	58 (67%)	< 0.001^b^
If yes, ILOS, median [IQR]	4 [2 – 10]	3 [2 – 6]	9 [4 – 17]	< 0.001^a^
Mechanically ventilated (at risk for VAP)	158 (20%)	102 (14%)	56 (65%)	< 0.001^b^
If yes, DMV, median [IQR]	2 [1 – 7]	2 [1 – 5]	5 [2 – 10]	< 0.001^a^
In-hospital mortality	23 (3%)	16 (2%)	7 (8%)	0.005^b^

Tests used to compare the subgroups: ^a^Wilcoxon rank-sum test; ^b^Chi-squared test; ^c^Fisher’s exact test

*AIS* Abbreviated Injury Scale, *DMV* days on mechanical ventilation, *ED* emergency department, *GCS* Glasgow Coma Scale, *IQR* interquartile range, *ISS* Injury Severity Score, *med* median, *MVC* motorized vehicle collison, *VAP* ventilator-associated pneumonia

Patients in the nosocomial pneumonia group were older and more often underwent blood product transfusion and emergent intubation than patients without pneumonia (all *p* < 0.001). Furthermore, the overall injury severity (ISS 9 vs. 22), the specific injury severity of the thorax (AIS thorax 0 vs. 3), and the head regions (GCS score 15 vs. 13) were higher in the nosocomial pneumonia group (all *p* < 0.001). All patients in the nosocomial pneumonia group suffered from blunt trauma compared to 97% of those who did not develop nosocomial pneumonia (Table 2).

### Original model and intercept recalibration

**Fig. 2:**
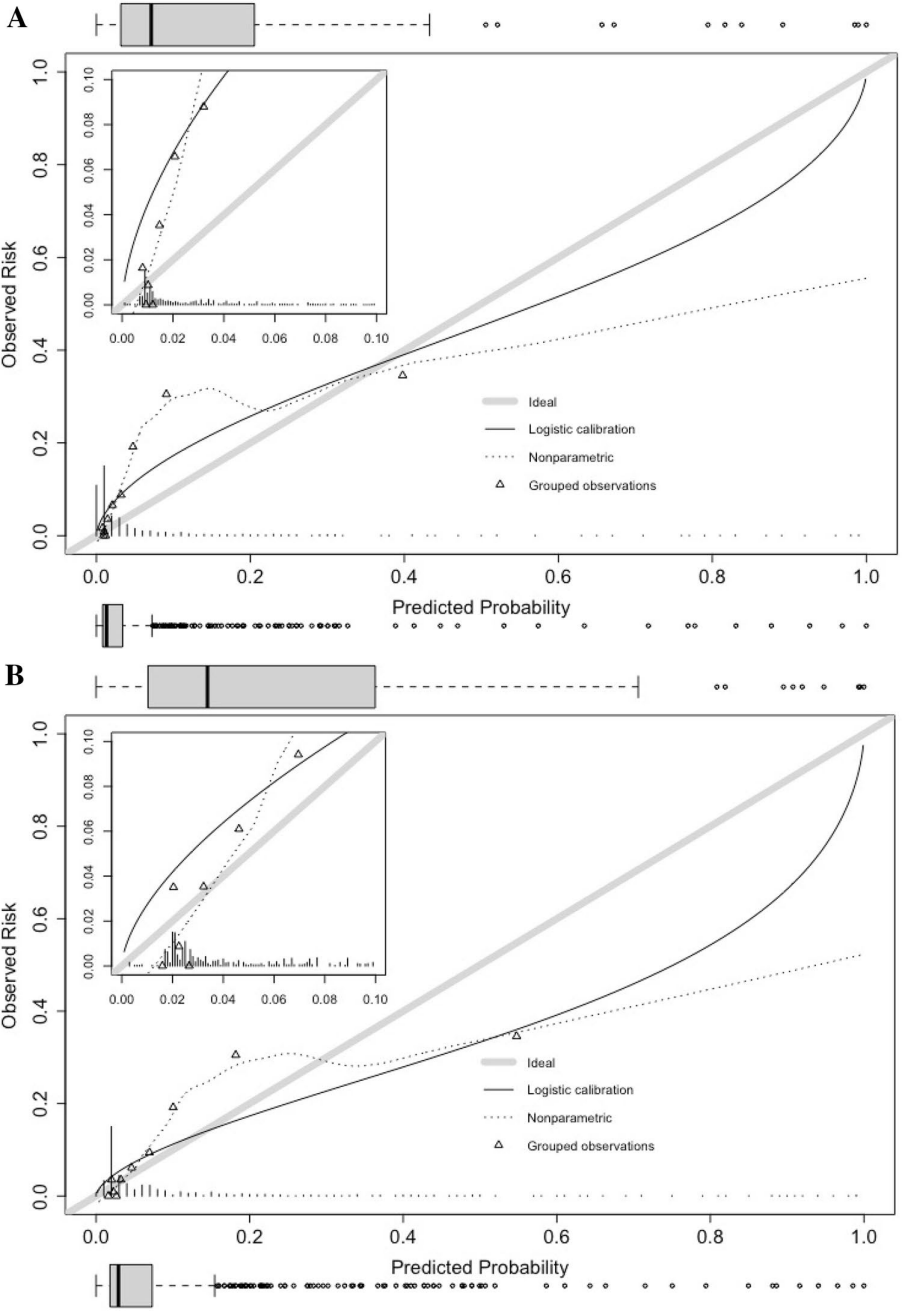
Calibration plots (slope = 0.609) of the original Croce formula in the Dutch 2017 cohort (**A**) and after intercept recalibration (**B**; slope = 0.609); zoomed perspectives are included in the left upper corners. Box plots for probability distribution are added per calibration plot for patients with (upper) and without (lower) nosocomial pneumonia

External validation of the Croce model showed an AUC of 0.83 (95% CI 0.79 – 0.87). The mean predicted probability of pneumonia was 6.4%, whereas the observed risk was 10.6%. The calibration slope was 0.63, suggesting some overfitting in the original study compared to our population. This overfitting resulted in predicted risks that were too extreme: the lower predicted probabilities were too low, while the higher predicted probabilities appeared to be too high (Fig. 2A). The model’s accuracy was good (Brier’s statistic 0.089; Supplemental Table 3).

**Table 3:** Logistic regression parameters for first model recalibration with nosocomial pneumonia as the outcome

Parameter	ORs	95% CI	*p* value
Mechanism of injury	NA	–	NA
GCS score	0.93	0.88 – 0.98	0.011
Spinal cord injury	0.90	0.22 – 3.09	0.878
AIS thorax	1.19	0.98 – 1.44	0.072
Emergency laparotomy	0.42	0.09 – 1.58	0.229
Blood products in ED	1.05	0.82 – 1.35	0.669
ISS	1.08	1.05 – 1.12	< 0.001
Emergency intubation	1.74	0.82 – 3.56	0.137

Mechanism of injury, blunt (0) or penetrating (1); Spinal cord injury, no (0) or yes (1); Emergency laparotomy, no (0) or yes (1); Blood products in ED, units; Emergency intubation, no (0) or yes (1)

*AIS* Abbreviated Injury Scale, *CI* confidence interval, *ED* emergency department, *GCS* Glasgow Coma Scale, *ISS* Injury Severity Score, *NA* not applicable, *OR* odds ratio

After intercept recalibration, the model’s intercept increased from -3.08 in the Croce model to 0.80 in our cohort, showing better calibration (Fig. 2B). Since only the intercept was corrected, the AUC remained the same.

### Model recalibration

**Fig. 3:**
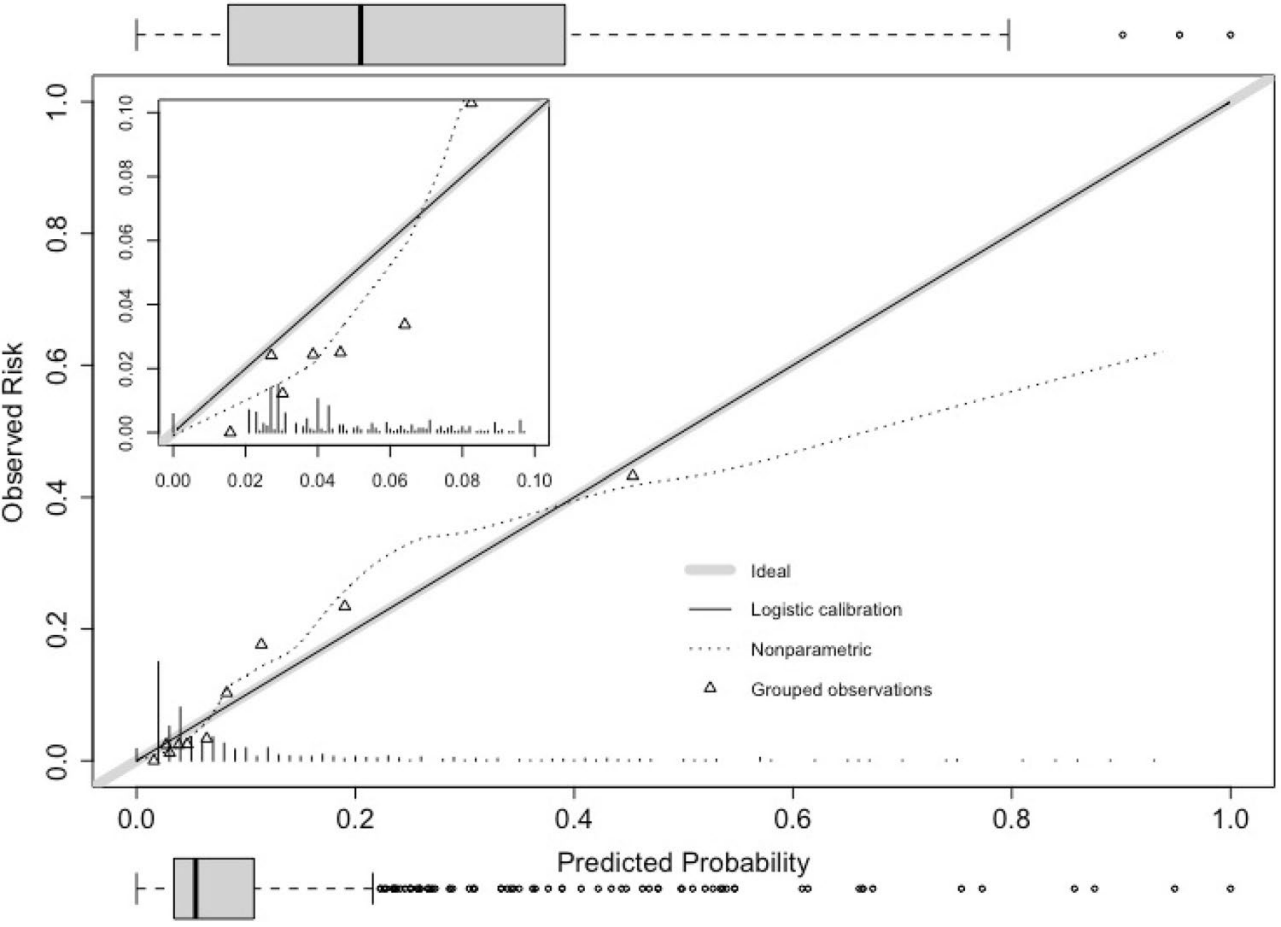
Calibration plot after the first model recalibration; a zoomed perspective is included in the left upper corner. Box plots for probability distribution are added for patients with (upper) and without (lower) nosocomial pneumonia

The first model recalibration slightly improved the discriminatory capacity (AUC 0.84, 95% CI 0.80 – 0.88) and showed an overall better concordance in calibration (Fig. 3). Brier’s statistic improved to 0.087 (Supplemental Table 3).

Since all pneumonia patients suffered from blunt trauma, the regression coefficient for mechanism of injury could not be estimated. A lower level of consciousness (odds ratio [OR] 0.93, 95% CI 0.88 – 0.98) and higher injury severity (OR 1.08, 95% CI 1.05 – 1.12) were independent predictors of pneumonia (Table 3).

### Additional analyses

In additional analyses, after the exclusion of mechanism of injury, the odds ratios of the other parameters remained similar and resulted in a comparable AUC of 0.84 (95% CI 0.79 – 0.88). Also, model calibration remained similar (Supplemental Table 2; Supplemental Fig. 2). In contrast, adding the variable age to the model improved the discriminatory capacity (AUC 0.87, 95% CI 0.84 – 0.91), and age appeared to be a good, independent predictor of nosocomial pneumonia (OR 1.03, 95% CI 1.02 – 1.05, Supplemental Table 1). The in-sample calibration of this model was good: more pneumonia cases were in the higher deciles, and the deciles were closer to the ideal line in the calibration plot (Supplemental Table 2; Supplemental Fig. 3). Moreover, the model’s accuracy improved further (Brier’s statistic 0.078; Supplemental Table 3).

After Ridge penalization, the AUC was similar for all additional analyses, indicating reliable discriminatory capacities (Supplemental Table 3). Since a penalty was introduced, the calibration slope became greater than 1, and predicted chances were pulled slightly toward the mean predicted probability.

### Discrimination for specific pneumonia entities

For each step, the discrimination of nosocomial pneumonia was better than that of the separate entities HAP and VAP. For each step, the model’s discrimination and accuracy for HAP were better than for VAP (Supplemental Table 3).

## Discussion

This study validated the Croce prediction model for post-traumatic nosocomial pneumonia in 809 patients admitted to a Dutch level-1 trauma center. The original model showed good discriminatory capacity (AUC 0.83, 95% CI 0.79 – 0.87) but was overfitted for use in the Dutch population (calibration slope 0.63; mean predicted risk 6.4% vs. 10.6% observed risk). This model and the intercept-recalibrated model can help in identifying high-risk patients. Full recalibration of the model improved the discriminatory capacity (AUC 0.84, 95% CI 0.80 – 0.88) and calibration in our study cohort. The recalibrated model provides a further basis for nosocomial pneumonia prediction in level-1 trauma patients. Patient monitoring of high-risk patients more rigorously might help clinicians recognize nosocomial pneumonia signs earlier. Furthermore, preventative actions such as pulmonary physical therapy or administering nebulized drugs could be initiated. We made use of several models for risk calculation accessible via an online tool (https://www.evidencio.com/) and Supplemental Table 4.

Croce’s cohort and our Dutch cohort of trauma patients were generally comparable and, therefore, seemed appropriate for external validation of the Croce model. The two cohorts of level-1 trauma patients were similar in the baseline characteristics of injury severity (i.e., ISS, level of consciousness, AIS thorax), emergent intubation, number of administered blood products, and spinal cord injury. Also, sex distribution was not substantially different (74% male vs. 67% in our cohort). However, in Croce’s cohort, the incidence of penetrating trauma was higher (23% vs. 3%), patients were younger (median 35 vs. 51 years), and relatively more patients underwent an emergent laparotomy (14% vs. 3%) compared to the Dutch cohort [6].

The incidence of nosocomial pneumonia was lower in Croce’s cohort than in the Dutch cohort (5.6% vs. 10.6%). This difference was most likely caused by expanding the primary outcome from VAP to nosocomial pneumonia. The proportion of VAP in our entire cohort was 6.2% (50 out of 809 patients), while in the patients at risk of VAP (*n* = 147), the proportion was 34.0%. In Croce’s predictor finding study, the incidence of post-traumatic pneumonia was 6% and consisted almost entirely (94.6%) of pneumonia in mechanically ventilated patients [5]. Furthermore, the age gap and the difference in trauma mechanism distribution between cohorts could explain the difference in nosocomial pneumonia incidence. Age was a strong predictor of nosocomial pneumonia in this study (OR 1.03 per year, 95% CI 1.02 – 1.05) and in previous research, and blunt trauma is a known risk factor for nosocomial pneumonia as well [2; 4–6; 9].

Ridge regression enabled the evaluation of the stability of individual predictors. The predictive performance of emergent laparotomy and spinal cord injury might be less in Dutch level-1 trauma patients, as they were unstable predictors: the ORs differed considerably before and after penalization. Notably, these predictors had the largest 95% CI in the Croce study [6]. Emergent laparotomy was performed more frequently in Croce’s cohort, possibly because all patients with penetrating abdominal injury underwent this procedure. In the Dutch cohort, the proportion of emergent laparotomies in penetrating trauma patients compared to blunt trauma patients was higher (21% vs. 6%). However, no penetrating trauma patients in our cohort developed pneumonia, potentially as few patients had penetrating trauma, penetrating injuries were often isolated, and penetrating injury patients were mostly young. Unpublished analyses showed that emergent laparotomy was performed more often in patients with a higher ISS and more severe thoracic and abdominal trauma. We hypothesize that the instability of emergent laparotomy as a predictor is three-fold. First, proper breathing is inhibited through postoperative pain. Second, ventilation after abdominal packing is mechanically regulated, which prevents physiologic responses to a starting pneumonia, such as increased work of breathing, coughing, and sighing. Third, emergent laparotomy is performed in penetrating trauma and severe blunt abdominal trauma. The first is performed more frequently, but the latter is a risk factor for nosocomial pneumonia [5, 6, 9].

Besides Croce et al., Cavalcanti et al. and Esnault et al. investigated whether spinal cord injury is a risk factor for VAP. Both studies found no association, which is concordant with the current results [3, 7]. Only four patients with spinal cord injury and six who underwent emergent laparotomy developed pneumonia in this study, leading to uncertainty in statistical analysis. Thoracic AIS score, overall injury severity, GCS score, and ED blood products had stable odds ratios after Ridge penalization. Their stability is congruent with previous research; all are known risk factors for nosocomial pneumonia [2, 7, 10, 13, 14, 18].

The predicted probabilities were low for all steps of the validation, recalibration, and sensitivity analyses (Supplemental Table 2). Initially, this resulted in an under-prediction of nosocomial pneumonia (mean predicted probability 6.8%). After the recalibration steps, optimization was mainly seen in the lower predicted probabilities, with few predicted probabilities above 0.25. Croce et al. considered patients with a predicted probability above 0.5 at risk of pneumonia. A comparable cutoff is not yet reasonable for Dutch level-1 trauma patients when using the recalibrated or updated model.

The difference between the models’ capability to predict HAP and VAP might indicate that selective digestive decontamination—generally administered to all ICU patients in the Netherlands—affected VAP development. Nonetheless, pneumonia occurred mostly in mechanically ventilated ICU patients (50 out of 86 nosocomial pneumonia cases). Hence, the effect of selective digestive decontamination on the prediction of nosocomial pneumonia in this study remains unknown. Future studies on nosocomial pneumonia prediction should aim to improve calibration and determine a workable cutoff value to facilitate implementing a nosocomial pneumonia prediction model. Model performance should be assessed in populations with and without selective digestive decontamination administration for prediction in hospitals that use this prophylaxis.

This study contains several additional limitations, in addition to the standard limitations that apply to retrospective research. First, the study outcome was expanded from VAP to nosocomial pneumonia. This expansion does not impact the study results as we recalibrated the model. Second, no standardized definition of pneumonia diagnosis was used. Also, the nosocomial pneumonia entities HAP and VAP were only briefly assessed, although selective digestive decontamination was administered to ICU patients. However, as this prophylaxis is the standard of care in hospitals in the Netherlands, using the total cohort for external validation is acceptable [16]. More extensive correction methods were not feasible because of the limited size of the study population. Third, relatively few patients had penetrating injuries or were diagnosed with spinal cord injuries, and the total sample size of this study was limited. Using Ridge penalization ensured that overfitting was unlikely, and the value of infrequent predictors could be assessed through their stability. Lastly, we did not correct for competing risks, except for patients who died within 48 h of admission. However, survival analysis would not have been suitable given this cohort’s relatively small number of events. Also, selection bias would vastly increase if patients who died without nosocomial pneumonia were excluded.

In conclusion, the externally validated and intercept-recalibrated models show good discrimination and have the potential to predict nosocomial pneumonia. At this time, clinicians could apply these models to identify high-risk patients, increase patient monitoring, and initiate preventative measures, such as pulmonary physical therapy or administering nebulized drugs. Our recalibrated models improve prediction in our study cohort but need external validation. We made several models available via an online tool. Ultimately, a model could enable patient-specific risk assessment and personalized decision-making. Larger studies in populations with and without selective digestive decontamination administration are needed to improve calibration and prediction further.

## Supplementary Information

Below is the link to the electronic supplementary material.

Supplementary file1 (DOCX 405 KB)

## Data Availability

The study data will not be made available.

## Abbreviations

AIS: Abbreviated Injury Scale
AUC: Area-under-the-receiver operating characteristic curve
CI: Confidence interval
ED: Emergency department
GCS: Glasgow Coma Scale
HAP: Hospital-acquired pneumonia
ICU: Intensive care unit
IQR: Interquartile range
ISS: Injury Severity Score
OR: Odds ratio
UMCU: University Medical Center Utrecht
VAP: Ventilator-associated pneumonia
