# Supplementary material for: A recalibrated prediction model can identify level-1 trauma patients at risk of nosocomial pneumonia
Source: Arch Orthop Trauma Surg. 2023 Jan 17;143(8):4933–41. doi: 10.1007/s00402-023-04766-5 (PMC10374678; doi:10.1007/s00402-023-04766-5)
Supplement: Supplementary file 1 — Supplementary file1 (DOCX 405 KB) [file 402_2023_4766_MOESM1_ESM.docx]

| **Supplemental Table 1.** Logistic regression and Ridge regression parameters for recalibrated models of nosocomial pneumonia in level-1 trauma patients. | | | | | |
| --- | --- | --- | --- | --- | --- |
|  | **Parameter** | **OR** | **95% CI** | ***p*-value** | **OR Ridge** |
| First model revision (all Croce parameters)^1^ | Mechanism of injury | NA | – | NA | 0.56 |
|  | GCS score | 0.93 | 0.88 – 0.98 | 0.011 | 0.93 |
|  | Spinal cord injury | 0.90 | 0.22 – 3.09 | 0.878 | 1.19 |
|  | AIS thorax | 1.19 | 0.98 – 1.44 | 0.072 | 1.19 |
|  | Emergent laparotomy | 0.42 | 0.09 – 1.58 | 0.229 | 0.84 |
|  | Blood products in ED | 1.05 | 0.82 – 1.35 | 0.669 | 1.10 |
|  | ISS | 1.08 | 1.05 – 1.12 | <0.001 | 1.05 |
|  | Emergent intubation | 1.74 | 0.82 – 3.56 | 0.137 | 1.56 |
|  |  |  |  |  |  |
| Second full model recalibration (all Croce parameters without mechanism of injury)^2^ | GCS score | 0.93 | 0.88 – 0.98 | 0.011 | 0.93 |
|  | Spinal cord injury | 0.91 | 0.22 – 3.11 | 0.885 | 1.19 |
|  | AIS thorax | 1.19 | 0.98 – 1.44 | 0.074 | 1.19 |
|  | Emergent laparotomy | 0.41 | 0.09 – 1.48 | 0.204 | 0.80 |
|  | Blood products in ED | 1.05 | 0.82 – 1.34 | 0.699 | 1.09 |
|  | ISS | 1.09 | 1.05 – 1.12 | <0.001 | 1.05 |
|  | Emergent intubation | 1.78 | 0.83 – 3.64 | 0.122 | 1.58 |
|  |  |  |  |  |  |
| Third full model recalibration (all Croce parameters without mechanism of injury and with age)^3^ | GCS score | 0.91 | 0.86 – 0.97 | 0.004 | 0.93 |
|  | Spinal cord injury | 0.71 | 0.16 – 2.64 | 0.627 | 1.17 |
|  | AIS thorax | 1.21 | 1.00 – 1.47 | 0.051 | 1.18 |
|  | Emergent laparotomy | 0.54 | 0.11 – 2.13 | 0.412 | 0.95 |
|  | Blood products in ED | 1.03 | 0.80 – 1.32 | 0.804 | 1.10 |
|  | ISS | 1.10 | 1.06 – 1.14 | <0.001 | 1.04 |
|  | Emergent intubation | 1.54 | 0.71 – 3.18 | 0.257 | 1.49 |
|  | Age | 1.03 | 1.02 – 1.05 | <0.001 | 1.01 |
| **Ridge penalties:** ^1^lambda = 0.0695; ^2^lambda = 0.0633; ^3^lambda = 0.0918. | | | | | |
| **Abbreviations**: AIS, Abbreviated Injury Scale; CI, confidence interval; ED, Emergency Department; GCS, Glasgow Coma Scale; ISS, Injury Severity Score; NA, not applicable; OR, odds ratio; | | | | | |

| **Supplemental Table 2.** Distribution of nosocomial pneumonia chance proportions divided into deciles of equal size. | | | | | | | | |
| --- | --- | --- | --- | --- | --- | --- | --- | --- |
|  |  | Conventional logistic regression model | | |  | Logistic regression model using Ridge penalization | | |
|  | Decile | Highest predicted probability in decile | Group size | PN+  (cumulative %) |  | Highest predicted probability in decile | Group size | PN+  (cumulative %) |
| External validation | **1** | 0.009 | 122 | 2 (2%) |  | NA | | |
|  | **2** | 0.010 | 61 | 1 (3%) |  |  |  |  |
|  | **3** | 0.011 | 60 | 0 (3%) |  |  |  |  |
|  | **4** | 0.013 | 89 | 1 (5%) |  |  |  |  |
|  | **5** | 0.017 | 74 | 2 (7%) |  |  |  |  |
|  | **6** | 0.027 | 82 | 5 (13%) |  |  |  |  |
|  | **7** | 0.036 | 85 | 8 (22%) |  |  |  |  |
|  | **8** | 0.062 | 75 | 14 (38%) |  |  |  |  |
|  | **9** | 0.146 | 80 | 25 (67%) |  |  |  |  |
|  | **10** | 1.000 | 81 | 28 (100%) |  |  |  |  |
|  |  |  |  |  |  |  |  |  |
| Recalibration in the large | **1** | 0.020 | 122 | 2 (2%) |  | NA | | |
|  | **2** | 0.021 | 61 | 1 (3%) |  |  |  |  |
|  | **3** | 0.025 | 60 | 0 (3%) |  |  |  |  |
|  | **4** | 0.029 | 89 | 1 (5%) |  |  |  |  |
|  | **5** | 0.037 | 74 | 2 (7%) |  |  |  |  |
|  | **6** | 0.059 | 82 | 5 (13%) |  |  |  |  |
|  | **7** | 0.078 | 85 | 8 (22%) |  |  |  |  |
|  | **8** | 0.129 | 75 | 14 (38%) |  |  |  |  |
|  | **9** | 0.276 | 80 | 25 (67%) |  |  |  |  |
|  | **10** | 1.000 | 81 | 28 (100%) |  |  |  |  |
|  |  |  |  |  |  |  |  |  |
| First model revision^1^ | **1** | 0.025 | 84 | 0 (0%) |  | 0.043 | 82 | 0 (0%) |
|  | **2** | 0.030 | 132 | 3 (3%) |  | 0.045 | 116 | 3 (3%) |
|  | **3** | 0.034 | 27 | 0 (3%) |  | 0.052 | 56 | 0 (3%) |
|  | **4** | 0.041 | 82 | 2 (6%) |  | 0.056 | 78 | 1 (5%) |
|  | **5** | 0.054 | 80 | 2 (8%) |  | 0.067 | 79 | 2 (7%) |
|  | **6** | 0.072 | 89 | 3 (12%) |  | 0.087 | 74 | 4 (12%) |
|  | **7** | 0.097 | 82 | 9 (22%) |  | 0.107 | 93 | 10 (23%) |
|  | **8** | 0.140 | 71 | 13 (37%) |  | 0.136 | 69 | 13 (38%) |
|  | **9** | 0.251 | 81 | 19 (59%) |  | 0.210 | 81 | 20 (62%) |
|  | **10** | 0.938 | 81 | 35 (100%) |  | 0.867 | 81 | 33 (100%) |
|  |  |  |  |  |  |  |  |  |
|  |  |  |  |  |  |  |  |  |
|  |  |  |  |  |  |  |  |  |
|  |  |  |  |  |  |  |  |  |
| Second full model recalibration (all Croce parameters without mechanism of injury)^2^ | **1** | 0.026 | 86 | 0 (0%) |  | 0.041 | 126 | 2 (2%) |
|  | **2** | 0.029 | 119 | 3 (3%) |  | 0.043 | 65 | 1 (3%) |
|  | **3** | 0.034 | 38 | 1 (5%) |  | 0.050 | 56 | 0 (3%) |
|  | **4** | 0.042 | 86 | 2 (7%) |  | 0.055 | 81 | 1 (5%) |
|  | **5** | 0.054 | 80 | 1 (8%) |  | 0.066 | 80 | 2 (7%) |
|  | **6** | 0.069 | 78 | 3 (12%) |  | 0.088 | 77 | 6 (14%) |
|  | **7** | 0.094 | 84 | 9 (22%) |  | 0.106 | 91 | 8 (23%) |
|  | **8** | 0.142 | 76 | 13 (37%) |  | 0.138 | 71 | 14 (40%) |
|  | **9** | 0.247 | 82 | 20 (60%) |  | 0.210 | 81 | 19 (62%) |
|  | **10** | 0.936 | 80 | 34 (100%) |  | 0.872 | 81 | 33 (100%) |
|  |  |  |  |  |  |  |  |  |
| Third full model recalibration (all Croce parameters without mechanism of injury and with age)^3^ | **1** | 0.011 | 81 | 0 (0%) |  | 0.038 | 81 | 0 (0%) |
|  | **2** | 0.017 | 81 | 0 (0%) |  | 0.046 | 82 | 0 (0%) |
|  | **3** | 0.027 | 81 | 0 (0%) |  | 0.053 | 80 | 1 (1%) |
|  | **4** | 0.037 | 81 | 2 (2%) |  | 0.061 | 81 | 0 (1%) |
|  | **5** | 0.051 | 81 | 0 (2%) |  | 0.074 | 81 | 3 (5%) |
|  | **6** | 0.071 | 80 | 4 (7%) |  | 0.089 | 80 | 4 (9%) |
|  | **7** | 0.097 | 81 | 8 (16%) |  | 0.112 | 81 | 8 (19%) |
|  | **8** | 0.157 | 81 | 15 (34%) |  | 0.143 | 81 | 12 (33%) |
|  | **9** | 0.265 | 81 | 22 (59%) |  | 0.209 | 81 | 23 (59%) |
|  | **10** | 0.956 | 81 | 35 (100%) |  | 0.840 | 81 | 35 (100%) |
| **Ridge penalties:** ^1^lambda = 0.0695; ^2^lambda = 0.0633; ^3^lambda = 0.0918. | | | | | | | | |
| **Abbreviations**: NA, not applicable; PN+, number of observed nosocomial pneumonia cases | | | | | | | | |

| **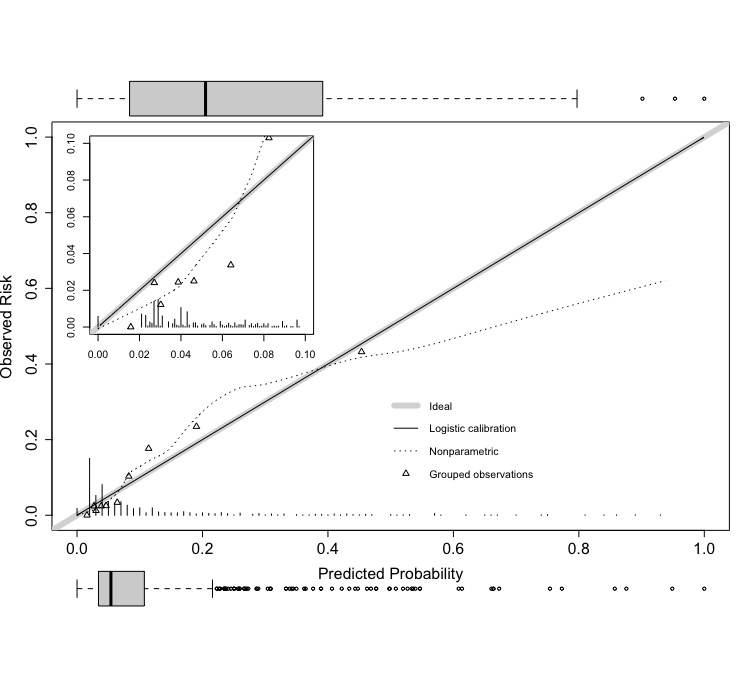A** |
| --- |
| **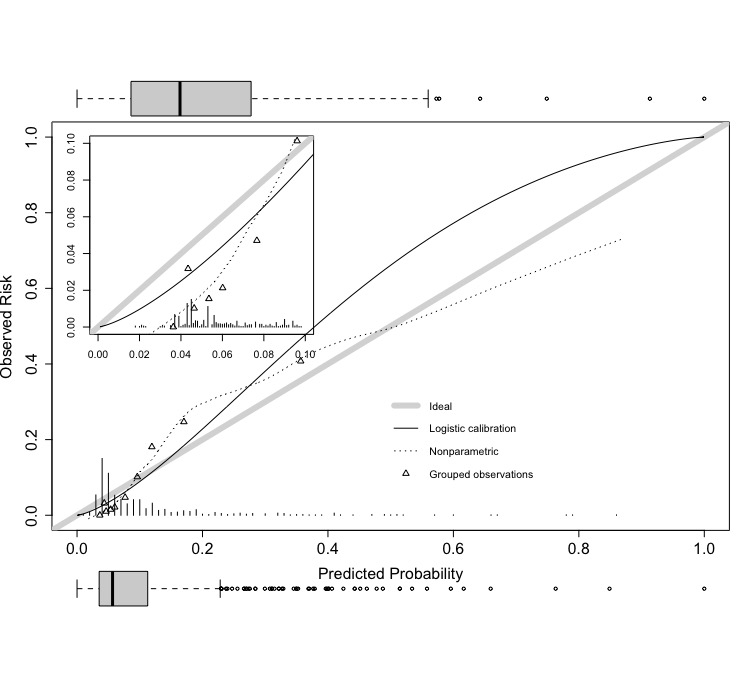B** |
| **Supplemental Figure 1**. Calibration plots (slope_A_ = 1.00; slope_B_ = 1.362) of the first model recalibration with (**B**) and without (**A**) Ridge regression analysis correction (lambda = 0.0695); zoomed perspectives are included in the left upper corners. Boxplots for probability distribution are added per calibration plot for patients with (upper) and without (lower) nosocomial pneumonia. |

| **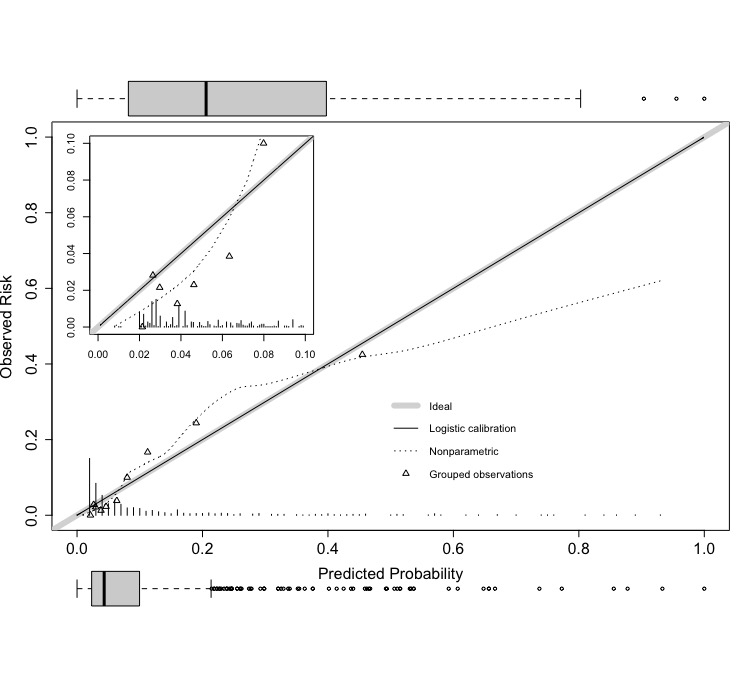A** |
| --- |
| **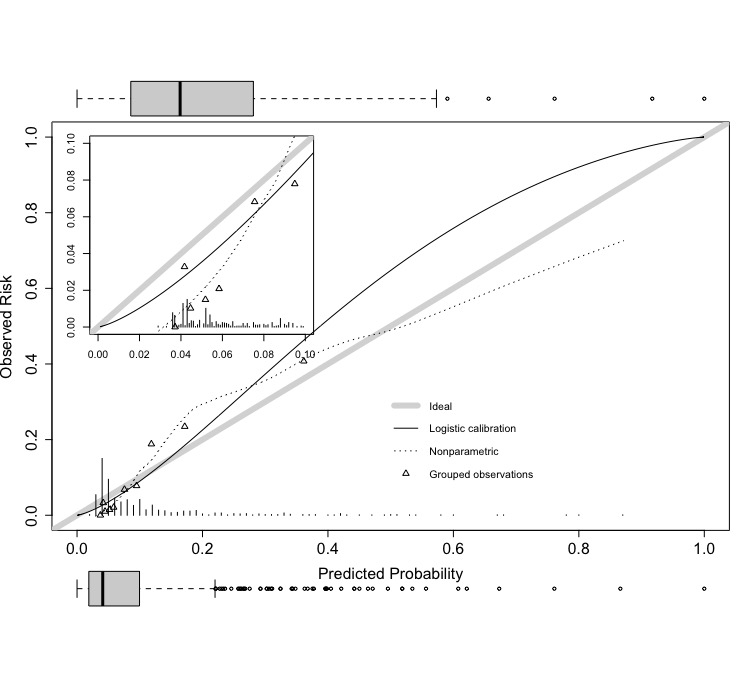B** |
| **Supplemental Figure 2**. Calibration plots (slope_A_ = 1.00; slope_B_ = 1.329) of the second model recalibration (without mechanism of injury) with (**B**) and without (**A**) Ridge regression analysis correction (lambda = 0.0633); zoomed perspectives are included in the left upper corners. Boxplots for probability distribution are added per calibration plot for patients with (upper) and without (lower) nosocomial pneumonia. |

| **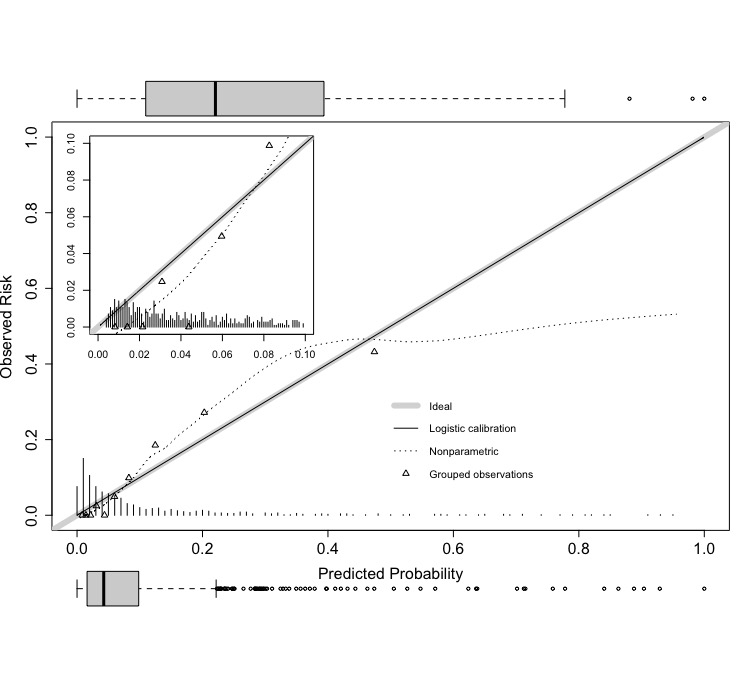A** |
| --- |
| **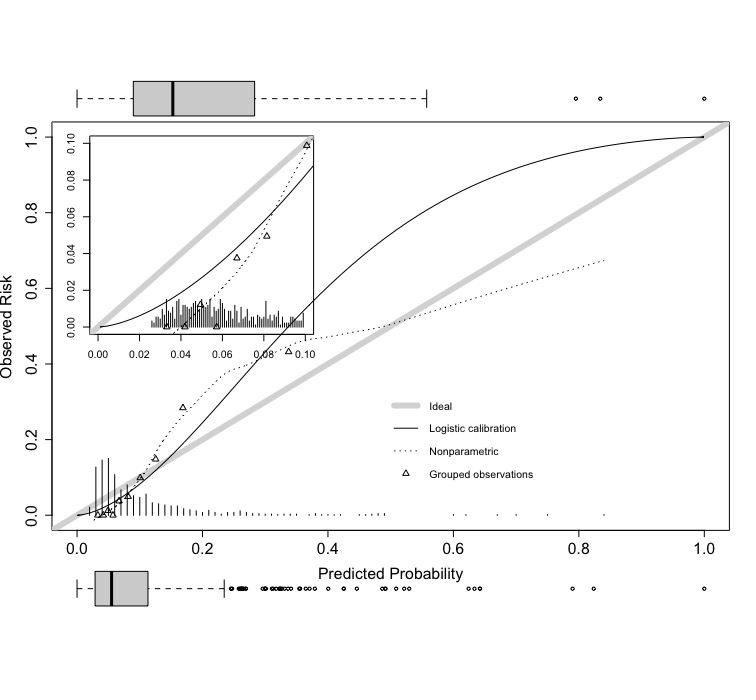B** |
| **Supplemental Figure 3.** Calibration plots (slope_A_ = 1.00; slope_B_ = 1.581) of the third model recalibration (without mechanism of injury and with age) with (**B**) and without (**A**) Ridge regression analysis correction (lambda = 0.0918); zoomed perspectives are included in the left upper corners. Boxplots for probability distribution are added per calibration plot for patients with (upper) and without (lower) nosocomial pneumonia. |

| **Supplemental Table 3.** AUCs (95% CIs) and Brier’s statistics for all validation and recalibration steps. The statistics were calculated for nosocomial pneumonia, hospital-acquired pneumonia, and ventilator-associated pneumonia. | | | | | | | | | |
| --- | --- | --- | --- | --- | --- | --- | --- | --- | --- |
|  |  | Nosocomial pneumonia *(n = 809)* | |  | Hospital-acquired pneumonia *(n = 662)* | |  | Ventilator-associated pneumonia *(n = 147)* | |
|  |  | No Ridge | Ridge |  | No Ridge | Ridge |  | No Ridge | Ridge |
| External validation | AUC | 0.831 (0.790 – 0.873) | NA |  | 0.791 (0.714 – 0.868) | NA |  | 0.605 (0.510 – 0.700) | NA |
|  | Brier | 0.089 |  |  | 0.050 |  |  | 0.266 |  |
| Recalibration in the large | AUC | 0.831 (0.790 – 0.873) |  |  | 0.791 (0.714 – 0.868) |  |  | 0.605 (0.510 – 0.700) |  |
|  | Brier | 0.087 |  |  | 0.050 |  |  | 0.254 |  |
| First model revision^1^ | AUC | 0.840 (0.797 – 0.882) | 0.838 (0.796 – 0.880) |  | 0.795 (0.719 – 0.870) | 0.793 (0.716 – 0.869) |  | 0.728 (0.648 – 0.809) | 0.727 (0.646 – 0.807) |
|  | Brier | 0.080 | 0.080 |  | 0.048 | 0.048 |  | 0.199 | 0.200 |
| Second model revision^2^ | AUC | 0.836 (0.793 – 0.879) | 0.836 (0.793 – 0.878) |  | 0.786 (0.708 – 0.865) | 0.788 (0.710 – 0.866) |  | 0.727 (0.646 – 0.808) | 0.727 (0.646 – 0.808) |
|  | Brier | 0.080 | 0.080 |  | 0.049 | 0.048 |  | 0.199 | 0.201 |
| Third model revision^3^ | AUC | 0.874 (0.842 – 0.906) | 0.867 (0.834 – 0.900) |  | 0.862 (0.817 – 0.906) | 0.849 (0.799 – 0.899) |  | 0.769 (0.693 – 0.845) | 0.767 (0.691 – 0.843) |
|  | Brier | 0.078 | 0.079 |  | 0.048 | 0.048 |  | 0.187 | 0.191 |
| **Ridge penalties:** ^1^lambda = 0.0695; ^2^lambda = 0.0633; ^3^lambda = 0.0918. | | | | | | | | | |
| **Abbreviations**: AUC, area-under-the-receiver operating characteristic curve; CI, confidence interval; NA, not applicable. | | | | | | | | | |

| **Supplemental Table 4.** The intercept-recalibrated model (Formula 1) and fully recalibrated model (Formula 2; without mechanism of injury and with age) to calculate nosocomial pneumonia risk in level-1 trauma patients. | |
| --- | --- |
| $f\left( x \right)= -0.80-1.56 \left( MOI \right)-0.12 \left( GCS \right)+1.37 \left( SCI \right)+0.30 \left( AIS thorax \right)+1.87 \left( lap \right)+ 0.67 \left( tx \right)+0.05 \left( ISS \right)+0.66 \left( int \right)$ | (Formula 1) |
| $f\left( x \right)= -4.61-0.09 \left( GCS \right)-0.35 \left( SCI \right)+0.19 \left( AIS thorax \right)-$  $0.62 \left( lap \right)+0.03 \left( tx \right)+0.09 \left( ISS \right)+0.43 \left( int \right)+0.03 (age)$ | (Formula 2) |
| $P\left( pneumonia=1 \right\vert x)=\frac{1}{1+ e^{-f(x)}}$ | (Formula 3) |
| **Dichotomous variables**: MOI, blunt (0) or penetrating (1); SCI, yes (1) or no (0); lap, yes (1) or no (0); int, yes (1) or no (0). | |
| **Abbreviations**: AIS, Abbreviated Injury Scale; GCS, Glasgow Coma Scale score; int, emergency intubation; ISS, Injury Severity Score; lap, emergency laparotomy; MOI, mechanism of injury; P_pneumonia_, predicted probability of pneumonia development; SCI, spinal cord injury; tx, number of blood products administered. | |
